# Association of the consumption of common drinks with early puberty in both sexes

**DOI:** 10.3389/fpubh.2022.854477

**Published:** 2022-12-02

**Authors:** Meng-Che Tsai, Yungling Leo Lee, Yang Ching Chen

**Affiliations:** ^1^Department of Pediatrics, National Cheng Kung University Hospital, College of Medicine, National Cheng Kung University, Tainan, Taiwan; ^2^Institute of Biomedical Sciences, Academia Sinica, Taipei, Taiwan; ^3^College of Public Health, China Medical University, Taichung, Taiwan; ^4^Graduate Institute of Clinical Medicine, Taipei Medical University, Taipei, Taiwan; ^5^Department of Family Medicine, Taipei Medical University Hospital, Taipei, Taiwan; ^6^Department of Family Medicine, School of Medicine, College of Medicine, Taipei Medical University, Taipei, Taiwan; ^7^School of Nutrition and Health Sciences, College of Nutrition, Taipei Medical University, Taipei, Taiwan; ^8^Graduate Institute of Metabolism and Obesity Sciences, Taipei Medical University, Taipei, Taiwan

**Keywords:** early puberty, sugar-sweetened beverages, probiotics, added sugar, menarche, voice breaking, category of study: a population study

## Abstract

**Background:**

We examined the effect of sugar-sweetened beverages (SSB) and common drink intake on pubertal development in both sexes.

**Methods:**

Data were retrieved from Taiwan Children Health Study, which involved detailed pubertal stage assessments of 2,819 schoolchildren aged 11 years in 2011–2012. Drawings of secondary sexual characteristics and self-reported age at menarche or voice breaking were used to assess pubertal stages. Dietary intake was assessed using a detailed semi-quantitative food frequency questionnaire. Generalized estimating equation modeling was applied to obtain odds ratios (ORs) and 95% confidence intervals (CIs) to represent the effects of each drink on early pubertal development outcomes.

**Results:**

In boys, an one cup/day increment of a SSB was associated with earlier voice breaking (β = −0.12; 95% CI = −0.20, −0.04), whereas consuming yogurt (≥2 cups/day) was a protective factor against early puberty (OR = 0.78; 95% CI = 0.73, 0.83). In girls, SSB consumption was associated with increased risk of early puberty in a dose–response manner, and a similar protective effect of yogurt consumption and fermented probiotic drink (≥2 cups/day) against early puberty was observed (OR = 0.96; 95% CI = 0.94, 0.99). Furthermore, the intake of both total sugar and added sugar within SSBs increased risk of early puberty in girls but not in boys.

**Conclusions:**

Sugar-sweetened beverages were associated with early puberty, and probiotic drinks appeared to mitigate this link. These findings indicate that the gut–brain axis could play a crucial role in sexual maturation.

## Introduction

Puberty refers to the process of physical changes under the cascade of endocrine actions. It is generally regarded as a hallmark of sexual maturation (1). The pubertal progression from the initiation to full maturation varies, which implies a complex interplay of nutrition, psychological condition, socioeconomic status, and hormonal actions in an individual (2, 3). Early pubertal timing, notably defined by early age at menarche in females, has been extensively linked to adverse health outcomes, such as metabolic syndrome (4), fatty liver disease (5), cardiovascular diseases (6), and breast cancer (7). In addition to physical impacts, adolescents who mature early are disadvantaged by potential psychosocial maladaptation and are thus prone to depression and engaging in delinquent behavior, early sexual activity, as well as substance use (8–11). As the trend toward earlier pubertal timing becomes clearer (12–14), the gap between physical and psychological maturity toward the full social status of adulthood continues to lengthen (15). Taiwan is no exception, with a constantly falling age at menarche and an almost 2-year drop over three generations (16). Researchers and clinicians have continued their pursuit of the determinants of sexual maturation to address the adverse health effects associated with early maturation.

The intake of sugar-sweetened beverages (SSBs) has been associated with pro-glycemic traits, such as higher fasting glucose and fasting insulin levels (17), and a higher health risk of obesity and diabetes among adolescents (18). In total, 88.7% of Taiwanese adolescents consume more than one SSB every week (19), at an average of 446 ml per day (20). Along with the obesogenic trends in dietary habits, the consistent finding that girls with a higher bone mass index (BMI) tend to mature early supports the vital role of nutritional factors in pubertal timing (21, 22). Therefore, prior mechanistic discussions surrounding the link between SSB intake and early puberty generally focus on higher BMI (i.e., positive energy balance) as a strong driver of menarche. In addition to the effects *via* weight status, SSB consumption is independently associated with earlier age at menarche in a large prospective US study (23), even after participants' BMI was adjusted for. Pathways thought to be mediating mechanisms include the intake of high-glycemic foods, such as SSBs, resulting in a rapid and immediate increase in circulating insulin concentrations, which could upregulate insulin-like growth factor-1 (IGF-1), a hormone involved in the initiation of menarche *via* modulating the reproductive system through widespread effects on hypothalamus, pituitary, and ovaries (24). Specifically, serum levels of IGF-1 may be inversely associated with levels of sex hormone binding globulin (SHBG) in obese children, while gender difference is well noted in the association between adiposity related declines in SHBG and pubertal age in an earlier longitudinal study (25). Despite substantial evidence on girls, little is known about whether SSB intake is related to boys' sexual maturation, independent of weight status.

The mediating mechanism *via* SHBG and bioavailable sex steroids on pubertal onset has also been observed for other macronutrients (26). Non-sugar contents of food intakes are reported to be differentially associated with age at menarche (27–29). For instance, higher consumption of dietary fibers and vegetable proteins are associated with later age at menarche (27, 28), while higher consumption of animal proteins and poly-unsaturated fatty acids are associated with earlier age at menarche (29). The complexity of food contents also applied to dairy products that are composed of wide-ranging nutrients, thus contributing to inconsistent results of the association with age at menarche (30, 31). As there is surge in research on the physiological effects of intestinal microbiota, yogurts that are rich in probiotics have been found to have a protective effect against early breast development and menarche in a group of Chilean girls (31). However, their finding was not replicated in Iranian female samples (30). Evidence of the association between nutrition and pubertal timing in boys is even relatively scant. If nutritional factors have a direct mechanistic effect on pubertal timing, this may provide an actionable opportunity to prevent off-time puberty by manipulating pre-pubertal dietary practices.

To obtain epidemiological evidence on the relationship between SSB intake and pubertal timing in both sexes, it is imperative to prospectively survey the relationship between detailed SSB intake among growing adolescents and the risk of early puberty. We hypothesize that SSB intake is associated with pubertal timing in a gender dependent manner. Therefore, in this study, we examined the association between SSBs and several other types of drinks, with a particular focus on probiotics drinks, and longitudinal pubertal outcomes in a representative nationwide sample. Furthermore, we explored the effects of total sugar or added sugar in SSBs and other drinks on the risk of early puberty.

## Materials and methods

### Study design and participants

**Figure 1:**
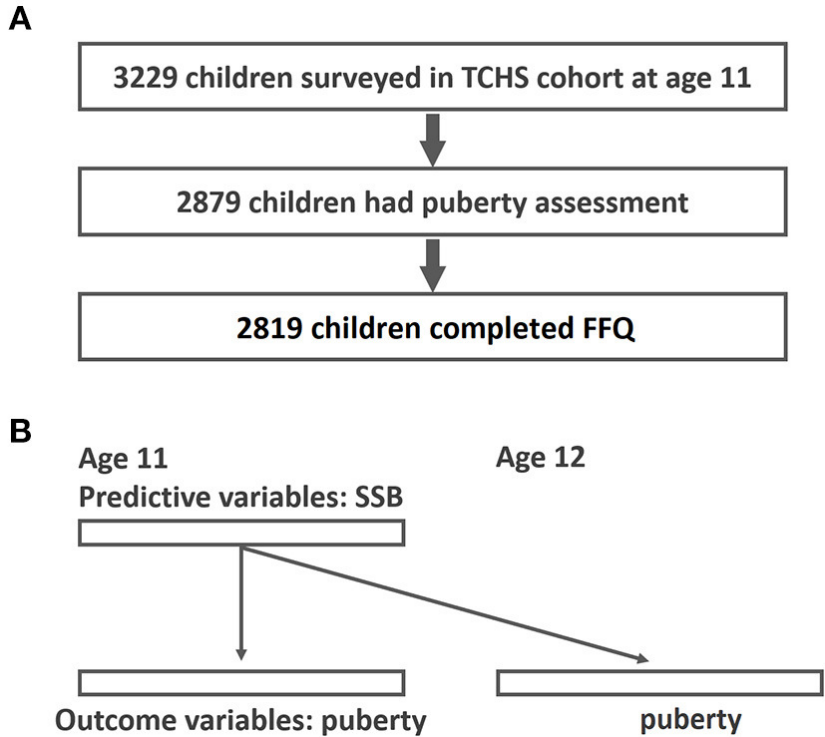
Study flow diagram of participant recruitment and GEE analysis for the associations of SSB and early puberty. **(A)** Study flow; **(B)** GEE analysis-time lag model of the associations between SSB and early puberty (predictive variables were SSB at age 11 and outcome variables were puberty outcomes at age 11 and age 12).

For this study, data were obtained from the Taiwan Children Health Study (TCHS), a prospective, nationwide cohort study focusing on obesity, pubertal development, and atopic diseases in adolescents. The TCHS had an open-cohort study design and comprised 3,229 children at age 11. The cross-sectional analysis herein mainly involved 2,819 schoolchildren aged 11–12 years in 2011–2012 with FFQ assessment and pubertal outcomes available (Figure 1). The detailed data collection in the TCHS was previously documented (32). Data regarding pubertal development were collected at age 11, 12, and 18. In addition, a food frequency questionnaire (FFQ) was administered at age 11. All participants received inform consent to participate in this study. The study protocol was approved by the Institutional Review Board of National Taiwan University Hospital and complied with the principles of the Declaration of Helsinki.

### Puberty outcomes

Puberty oucomes were assessed at age 11, 12, and 18. We used two types of children self-filled questionnaire to define pubertal outcomes: the Chinese version of the puberty development scale (PDS) (33) and the Tanner derived composite stage (TDCS) (34). Early puberty outcome was determined by the TDCS, and age at menarche or voice breaking was assessed by PDS. The TDCS contains drawings of the five stages of secondary sexual characteristics (pubic hair and breast development for girls and pubic hair and genital development for boys). The two sex characteristics (pubic hair for both genders, genitalia for boys and breast development for girls) were combined and averaged to create a single Tanner score (33). Furthermore, early puberty was defined as reaching a certain pubertal stage earlier than the median age for that stage (35), referencing large-scale population-based Chinese studies (36, 37). We validated the Chinese version of the TDCS, and the consistency between Tanner stages from children self-reports and physical inspection by pediatric endocrinologist was revealed to be high in our pilot study (weighted kappa = 0.85) (38). Age at menarche and age at voice breaking were defined according to questions in the PDS at age 11–12. Most of the children had completed pubertal growth at age 18, therefore, age at menarche and age at voice breaking were confirmed again using the PDS through participants' recall.

### Dietary assessment

We assessed diet using a self-administered, semi-quantitative 71-food-item FFQ in 2011 (39). It was a modified and shorter version of the Nutrition and Health Survey of Taiwan (40) and had been used in adolescents in one previous study (41) to investigate SSB intake. The original Chinese FFQ used in nationwide survey of Nutrition intake had been proved reproducible and of high validity (39). Participating children were instructed by a research dietitian for 20 min regarding how the portion size of each food item was measured in the FFQ. Participants were asked how frequently (on average in a week), they consumed a typical portion size of a certain food during the preceding month. A typical serving size was specified as 240 ml (equal to one cup) for SSBs, fresh juice, fresh milk, flavored milk, soy milk, yogurt, and fermented probiotic drinks. SSBs include all carbonated soft drinks, sweetened milk tea, and all other sugar sweetened beverages, such as flavored milk and concentrated fruit juice. Fresh juice, fresh milk, soy milk, etc… which contained no added sugar, were not included in the SSB category. Fermented probiotic drinks included Yakult, fermented milk, and other probiotic drinks. The probiotics contents in those fermented probiotic drinks mainly included *Lactobacillus* spp. The common probiotics contents within the yogurt include *Streptococcus thermophiles, Bifidobacterium lactis, Lactobacillus bulgaricus, and Lactobacillus acidophilus*. Detailed contents of fermented probiotic drinks and yogurt are listed in Supplementary Table S3. The simple sugar content in each type of drink was calculated by using a Taiwanese food composition table as the nutrient database (Taipei, Taiwan) (42). Total sugars were classified as natural sugar and added sugar. We classified natural sugars as lactose and non-lactose. Non-lactose sugars included glucose, fructose, galactose, sucrose, and maltose. Added sugar was defined as any sugar added to the drink during its production. After summing up the sugar intake by frequency and portion size to produce grams per day, we then categorized distinct sugar intake from drinks by using percentiles (≤25, 25−75%, and ≥75%).

### Covariate assessment

Confounders in the statistical models were *a priori* confounders based on previous research (23) with data relevant to both puberty outcomes and SSBs. Body height was measured to the nearest 0.1 cm using a wall-mounted stadiometer (MAGATA, BW-120) at each school. BMI was calculated as weight divided by (height)^2^ [kg/m^2^]. Body fat was measured using a tetrapolar multi-frequency bioelectrical impedance analysis machine (IOI 353, Jawon Medical, Korea). Participants with missing covariate information were included in the model by using missing indicators (43). Moreover, all models were adjusted for BMI, parental educational level, family income, and household smoking.

### Data analysis

Continuous variables were expressed as mean ± standard deviation and categorical variables were expressed as frequencies and percentages. The correlations between basic demographics and different SSB and drink consumption groups were examined by chi-square test (frequency with percentage) and analysis of variance (mean with standard deviation). To evaluate the repeated measurements of pubertal outcomes at age 11 and 12, we used generalized estimating equations (GEEs) (44)-Time lag model, to examine the interrelations among SSB intake and pubertal outcomes (Figure 1B). The primary advantage of a GEE is that it accounts for within-individual variations. The correlation structure assumed for repeated measurements was a working exchangeable correlation. In the Time-lag Model, predictors (SSB at age 11) were modeled using data from surveys that preceded the outcome variables (puberty outcome at age 11 and 12). Additionally, the time-lag model accounts for the temporal sequence in a possible cause-and-effect relationship (45). In addition to selected covariates, we adjusted all enlisted beverages and total energy with the use of variance inflation factor to test the collinearity among variables. Odds ratios (OR) and 95% confidence intervals (CIs) represented the effect of each drink on early pubertal development outcomes. Trend testing was performed to present the dose responsiveness. Missing covariate information of the participants was also included in the model by using missing indicators (43). All tests were two-sided with a 5% significance level. All analyses were performed using R version 3.3.2 (R Foundation for Statistical Computing).

## Results

### Participants' characteristics

Among 2,819 children analyzed in this study (Table 1), 472 (16.7%) children consumed 0 cup/day of SSB, while 628 (22.3%) children consumed ≥3 cup/day. The majority (1,719 children, 61.0%) of the children consumed 1–2 cups of SSB per day. One hundred sixty-one males (11.5%) and 139 females (9.8%) experienced early puberty. Compared with female participants, males tended to consume more SSBs with nearly 25.9% drinking ≥3 cup/day. Those consuming high quantities of SSB (≥3 cup/day) were more likely to hail from households with higher exposure to cigarette smoking, less parental education, and lower family income. However, no difference in BMI, body fat, and physical activity levels was observed across different SSB consumption groups. Moreover, intake of total energy, carbohydrates, additional sugar, and additional fat increased significantly with higher consumption frequency of SSBs, indicating that unhealthy eating habits might accompany higher levels of SSB consumption. Furthermore, intake of protein decreased as SSB consumption increased, suggesting that appropriate protein intake might be replaced by SSBs. However, parental educational level, family income, and household smoking revealed significantly differences in distribution between three SSB consumption groups, while they were adjusted in all models.

**Table 1:** Baseline characteristics according to categories of sugar-sweetened beverages in Taiwanese children at 11 years (*N* = 2,819).

**Characteristics**		**Total**		**0 cup/day**		**1-2 cups/day**		≥**3 cups/day**		P value^*^
		**Mean (SD)** **or** ***N*** **(%)**		**Mean (SD)** **or** ***N*** **(%)**		**Mean (SD)** **or** ***N*** **(%)**		**Mean (SD)** **or** ***N*** **(%)**		
Age	Years	11.1	0.30	11.09	0.30	11.09	0.33	11.08	0.31	0.75
Sex	Male	1,403	49.8	217	15.5	823	58.7	363	25.9	<0.001
	Female	1,416	50.2	255	18.0	896	63.3	265	18.7	
Breast feeding	Yes	928	49.8	156	16.8	555	59.8	217	23.4	0.42
	No	935	50.2	150	16.0	586	62.7	199	21.3	
Birthweight	g	3,133.9	459.6	3,123.8	409.1	3,129.7	482.4	3,153.2	432.0	0.61
Gestational age	Weeks	38.6	2.4	38.44	2.3	38.60	2.5	38.50	2.4	0.50
Household cigarette smoke	Yes	1,387	49.3	185	13.3	839	60.5	363	26.2	<0.001
	No	1,427	50.7	287	20.1	875	61.3	265	18.6	
Parental education > college	Yes	1,448	51.4	305	21.1	891	61.5	252	17.4	<0.001
	No	1,371	48.6	167	12.2	828	60.4	376	27.4	
Family income >600,001	Yes	1,542	54.7	287	18.6	953	61.8	302	19.6	<0.001
	No	1,277	45.3	185	14.5	766	59.9	326	25.5	
Physical activity >moderate	Yes	390	29.5	158	16.97	549	58.9	224	24.1	0.06
	No	931	70.5	60	15.38	256	65.6	74	18.9	
Body mass index	kg/m^2^	19.3	3.8	19.1	3.7	19.3	3.8	19.4	3.9	0.34
Body fat	%	17.7	7.7	17.6	7.6	17.9	7.7	17.24	7.85	0.08
Early puberty	Yes	300	10.6	44	9.3	182	10.6	74	11.8	0.42
Boy	Yes	161	11.5	26	12.0	90	10.9	45	12.4	0.74
Girl	Yes	139	9.8	18	7.1	92	10.3	29	10.9	0.25
Age at voice breaking (Boy)	Years	12.8	2.0	12.9	2.1	12.9	2.0	12.7	2.1	0.62
Age at menarche (Girl)	Years	11.5	1.1	11.4^#^	1.3	11.6^‡^	1.0	11.4	0.9	0.003
Energy intake	kcal	1,997.3	371.7	1,852.2	326.2	2,001.0	371.2	2,106.1	369.7	<0.001
Carbohydrate	% of energy	54.6	3.9	54.0	4.0	54.4^‡^	3.7	55.4^†^	4.1	<0.001
Protein	% of energy	15.5	1.9	16.5^#^	1.8	15.5^‡^	1.8	15.0^†^	1.9	<0.001
Fat	% of energy	29.9	3.8	29.5^#^	4.1	30.1^‡^	3.7	29.6	3.9	0.01
Additional sugar	% of energy	12.1	5.1	9.2^#^	4.6	12.3^‡^	4.8	13.9^†^	5.3	<0.001
Additional fat	% of energy	12.6	4.4	11.1^#^	4.4	12.9	4.4	13.0^†^	4.4	<0.001
Sodium	g per 1,000 kcal	1.3	0.3	1.4^#^	0.4^#^	1.2^‡^	0.3	1.2	0.4	<0.001

The number of participants did not add up to the total number because of missing data.

SD indicates standard deviation.

* The chi-square test was used for categorical variables and ANOVA for numerical variables.

ANOVA post hoc analysis of Bonferroni correction to compare the statistical difference among groups for the main SSB exposures and puberty outcomes in this study.

# Indicates a significant difference between 1-2 cups/day and 0 cups/day.

† Indicates a significant difference between ≥3 cups/day and 0 cups/day.

‡ Implies a significant difference between ≥3 cups/day and 1-2 cups/day.

### The associations in boys

Using GEE modeling, we observed that a 1 cup/day increment of SSB intake was associated with voice breaking at an earlier age (β = −0.18; 95% CI =-0.29, −0.06) in male participants (Table 2). However, consuming yogurt (≥2 cups/day) was a protective factor against early puberty (OR = 0.78; 95% CI = 0.73, 0.83). By contrast, consuming fermented probiotic drinks was associated with voice breaking at an earlier age (β= −1.23; 95% CI = −3.19, −0.72). Although fresh milk seemingly did not influence the timing of puberty among boys, consuming 1 cup/day of flavored milk increased the risk of early puberty (OR = 1.05; 95% CI 1.00, 1.02) and was associated with voice breaking at an earlier age (β = −0.41; 95% CI = −0.81, −0.01).

**Table 2:** OR and regression coefficients for the associations between consumption of selected drinks and pubertal outcomes (boys).

**Category (*N*, %)**	**Early puberty**				**Age at voice breaking**			
	**Unadjusted model**		**Adjusted model**		**Unadjusted model**		**Adjusted model**	
	**OR (95% CI)**	**P value**	**OR (95% CI)**	**P value**	**β (95% CI)**	**P value**	**β (95% CI)**	**P value**
**Sugar-sweetened beverages**								
Rare/never (217, 15%)	Ref		Ref		Ref		Ref	
1-2 cups/day (823, 59%)	1.02 (0.98, 1.06)	0.46	0.99 (0.94, 1.05)	0.78	0.08 (−0.31, 0.46)	0.69	−0.06 (−0.54, 0.42)	0.82
≥3 cups/day (363, 26%)	1.06 (1.01, 1.11)	0.02	1.01 (0.95, 1.07)	0.86	−0.39 (−0.81, 0.03)	0.07	−0.57 (−1.13, −0.02)	0.05
*p* for trend		0.01		0.69		0.005		0.01
1 cup/day increment	1.01 (1.00, 1.02)	0.004	1.01 (0.99, 1.01)	0.86	−0.14 (−0.21, −0.07)	< 0.001	−0.18 (−0.29, −0.06)	< 0.01
**Fresh juice**								
Rare/never (76, 9%)	Ref		Ref		Ref		Ref	
1-2 cups/day (625, 72%)	1.02 (0.96, 1.09)	0.48	1.01 (0.95, 1.08)	0.72	0.72 (0.19, 1.25)	0.01	0.67 (0.04, 1.29)	0.02
≥3 cups/day (172, 20%)	0.97 (0.91, 1.05)	0.49	0.98 (0.90, 1.06)	0.58	0.59 (−0.04, 1.21)	0.06	0.68 (0.01, 1.34)	0.04
*p* for trend		0.17		0.38		0.37		0.17
1 cup/day increment	0.97 (0.92, 1.02)	0.22	0.95 (0.84, 1.08)	0.43	0.14 (−0.34, 0.61)	0.58	0.76 (−0.32, 1.84)	0.16
**Fresh milk**								
Rare/never (356, 41%)	Ref		Ref		Ref		Ref	
1-2 cups/day (448, 51%)	1.00 (0.96, 1.04)	0.90	0.99 (0.95, 1.03)	0.48	0.29 (−0.05, 0.63)	0.10	0.24 (−0.12, 0.59)	0.20
≥3 cups/day (69, 8%)	0.98 (0.91, 1.05)	0.50	0.97 (0.89, 1.05)	0.36	−0.03 (−0.71, 0.66)	0.93	0.03 (−0.66, 0.72)	0.94
*p* for trend		0.62		0.32		0.42		0.44
1 cup/day increment	1.00 (0.97, 1.02)	0.73	0.99 (0.96, 1.02)	0.38	−0.02 (−0.24, 0.2)	0.84	−0.03 (−0.27, 0.21)	0.83
**Flavored milk**								
Rare/never (682, 78%)	Ref		Ref		Ref		Ref	
1 cup/day (176, 20%)	1.05 (1.00, 1.10)	0.04	1.05 (1.00, 1.10)	0.047	−0.35 (−0.75, 0.04)	0.08	−0.41 (−0.81, −0.01)	< 0.05
≥2 cups/day (15, 2%)	0.92 (0.83, 1.03)	0.14	0.93 (0.80, 1.08)	0.21	−0.34 (−1.77, 1.08)	0.64	−0.16 (−1.55, 1.22)	0.81
*p* for trend		0.20		0.19		0.09		0.07
1 cup/day increment	1.01 (0.97, 1.05)	0.51	1.02 (0.98, 1.06)	0.35	−0.11 (−0.45, 0.23)	0.55	−0.08 (−0.44, 0.27)	0.61
**Soy milk**								
Rare/never (40, 5%)	Ref		Ref		Ref		Ref	
1 cup/day (822, 94%)	1.03 (0.95, 1.12)	0.50	1.03 (0.94, 1.13)	0.47	−0.56 (−1.29, 0.18)	0.14	−0.51 (−1.18, 0.15)	0.19
≥2 cups/day (11, 1%)	1.17 (0.94, 1.46)	0.15	1.18 (0.98, 1.43)	0.13	0.77 (−0.6, 2.14)	0.27	0.60 (−0.85, 2.05)	0.40
*p* for trend		0.19		0.17		0.56		0.63
1 cup/day increment	1.05 (1.00, 1.09)	0.04	1.05 (1.00, 1.11)	0.07	−0.12 (−0.51, 0.27)	0.52	−0.13 (−0.52, 0.26)	0.5
**Yogurt**								
Rare/never (445, 52%)	Ref		Ref		Ref		Ref	
1 cup/day (427, 48%)	0.99 (0.96, 1.03)	0.63	0.98 (0.95, 1.02)	0.39	0.36 (0.04, 0.69)	0.03	0.33 (−0.01, 0.67)	0.05
≥2 cups/day (1, 0.1%)	0.84 (0.82, 0.86)	< 0.001	0.78 (0.73, 0.83)	< 0.001	–	–	–	–
*p* for trend		0.59		0.34		0.03		0.05
1 cup/day increment	1.01 (0.95, 1.08)	0.74	1.01 (0.94, 1.08)	0.84	0.05 (−0.53, 0.63)	0.87	0.30 (−0.38, 0.97)	0.29
**Fermented probiotic drink**								
Rare/never (321, 37%)	Ref		Ref		Ref		Ref	
1-2 cups/day (548, 63%)	1.01 (0.97, 1.05)	0.54	1.02 (0.98, 1.06)	0.27	0.17 (−0.18, 0.51)	0.35	0.20 (−0.15, 0.56)	0.26
≥3 cups/day (4, 0.5%)	0.96 (0.76, 1.21)	0.75	1.00 (0.77, 1.30)	0.99	−1.85 (−2.69, −1.01)	< 0.001	−1.23 (−3.19, −0.72)	0.005
*p* for trend		0.64		0.32		0.91		0.62
1 cup/day increment	1.03 (0.99, 1.08)	0.12	1.05 (1.00, 1.10)	0.07	−0.21 (−0.53, 0.12)	0.26	−0.09 (−0.46, 0.27)	0.55

Adjusted models were adjusted by: BMI, energy intake, parental educational level, family income, and household smoking, other drinks (total cups in SSB, fresh juice, fresh milk, flavored milk, soy milk, yogurt, fermented probiotic drink).

### The associations in girls

In girls, SSB consumption was associated with an increased risk of early puberty in a dose–response manner (Table 3). Girls consuming 1 cup/day of yogurt or fermented probiotic drink exhibited an increased risk of early puberty compared with those who rarely consumed those drinks [OR = 1.03 (95% CI = 1.00, 1.05) for yogurt and OR = 1.16 (95% CI = 1.11, 1.33) for fermented probiotic drinks]. By contrast, yogurt consumption (≥2 cups/day) was a protective factor against early puberty (OR = 0.92; 95% CI = 0.89, 0.95). Similarly, consuming fermented probiotic drinks (≥2 cups/day) was associated with a reduced risk of early puberty (OR = 0.96; 95% CI = 0.94, 0.99) as well as a delayed age at menarche (β = 0.41; 95% CI = 0.24, 1.42).

**Table 3:** OR and regression coefficients for the associations between consumption of selected drinks and pubertal outcomes (girls).

**Category (*N*, %)**	**Early puberty**				**Age at menarche**			
	**Unadjusted model**		**Adjusted model**		**Unadjusted model**		**Adjusted model**	
	**OR (95% CI)**	**P value**	**OR (95% CI)**	**P value**	**β (95% CI)**	**P value**	**β (95% CI)**	**P value**
**Sugar-sweetened beverages**								
Rare/never (255, 14%)	Ref		Ref		Ref		Ref	
1-2 cups/day (896, 50%)	1.03 (1.01, 1.06)	0.004	1.03 (0.99, 1.06)	0.07	0.24 (0.07, 0.42)	0.01	0.11 (−0.06, 0.29)	0.21
≥3 cups/day (265, 35%)	1.05 (1.01, 1.08)	0.004	1.04 (1.00, 1.08)	0.049	0.02 (−0.17, 0.22)	0.81	−0.13 (−0.35, 0.09)	0.24
*p* for trend		0.02		0.11		0.35		0.05
1 cup/day increment	1.01 (1.00, 1.01)	0.08	1.00 (1.00, 1.01)	0.25	−0.01 (−0.05, 0.03)	0.56	−0.04 (−0.09, 0.01)	0.10
**Fresh juice**								
Rare/never (50, 5%)	Ref		Ref		Ref		Ref	
< 0.5 cups/day (734, 72%)	1.04 (1.00, 1.08)	0.06	1.04 (0.99, 1.10)	0.05	0.32 (−0.06, 0.70)	0.10	0.27 (−0.07, 0.61)	0.20
≥0.5 cups/day (230, 23%)	1.03 (0.99, 1.08)	0.17	1.03 (0.97, 1.09)	0.30	0.38 (−0.01, 0.78)	0.06	0.27 (−0.10, 0.63)	0.23
*p* for trend		0.75		0.81		0.10		0.49
1 cup/day increment	1.00 (0.97, 1.03)	0.93	0.97 (0.90, 1.05)	0.47	0.09 (−0.11, 0.29)	0.37	−0.06 (−0.49, 0.36)	0.77
**Fresh milk**								
Rare/never (372, 37%)	Ref		Ref		Ref		Ref	
1-2 cups/day (570, 56%)	1.01 (0.99, 1.04)	0.29	1.00 (0.98, 1.03)	0.53	−0.06 (−0.20, 0.07)	0.35	−0.10 (−0.24, 0.03)	0.15
≥3 cups/day (72, 7%)	1.00 (0.95, 1.04)	0.87	0.96 (0.91, 1.01)	0.11	0.14 (−0.12, 0.40)	0.29	−0.16 (−0.52, 0.19)	0.34
*p* for trend		0.59		0.44		0.94		0.13
1 cup/day increment	1.01 (0.99, 1.03)	0.28	1.00 (0.98, 1.02)	0.65	0.05 (−0.04, 0.13)	0.28	−0.08 (−0.20, 0.04)	0.23
**Flavored milk**								
Rare/never (793, 78%)	Ref		Ref		Ref		Ref	
1 cup/day (211, 21%)	0.99 (0.96, 1.01)	0.35	0.99 (0.96, 1.02)	0.36	0.10 (−0.06, 0.27)	0.23	0.11 (−0.05, 0.27)	0.20
≥2 cups/day (10, 1%)	1.17 (0.96, 1.41)	0.12	1.17 (0.94, 1.32)	0.17	−0.18 (−1.02, 0.67)	0.68	−0.61 (−1.32, 0.10)	0.21
*p* for trend		0.82		0.89		0.36		0.59
1 cup/day increment	1.02 (0.98, 1.06)	0.18	1.02 (0.99, 1.05)	0.44	0.01 (−0.17, 0.18)	0.92	−0.06 (−0.21, 0.08)	0.49
**Soy milk**								
Rare/never (73, 7%)	Ref		Ref		Ref		Ref	
1 cup/day (928, 92%)	1.02 (0.99, 1.06)	0.19	1.03 (0.98, 1.07)	0.20	0.11 (−0.14, 0.36)	0.39	0.06 (−0.22, 0.33)	0.67
≥2 cups/day (13, 1%)	1.09 (0.95, 1.26)	0.24	1.05 (0.94, 1.18)	0.50	0.31 (−0.34, 0.96)	0.35	0.02 (−0.67, 0.72)	0.94
*p* for trend		0.14		0.21		0.32		0.73
1 cup/day increment	1.03 (0.99, 1.07)	0.06	1.03 (0.99, 1.05)	0.33	0.08 (−0.09, 0.25)	0.37	−0.02 (−0.20, 0.16)	0.85
**Yogurt**								
Rare/never (429, 42%)	Ref		Ref		Ref		Ref	
1 cup/day (580, 57%)	1.03 (1.01, 1.05)	0.01	1.03 (1.00, 1.05)	0.03	0.14 (0.01, 0.27)	0.04	0.09 (−0.05, 0.22)	0.21
≥2 cups/day (5, 0.5%)	0.95 (0.94, 0.96)	< 0.001	0.92 (0.89, 0.95)	< 0.001	0.49 (0.38, 0.59)	< 0.001	0.20 (−0.64, 1.03)	0.07
*p* for trend		0.02		0.06		0.02		0.19
1 cup/day increment	1.02 (0.98, 1.06)	0.34	1.00 (0.96, 1.04)	0.99	0.12 (−0.04, 0.27)	0.28	−0.03 (−0.26, 0.20)	0.75
**Fermented probiotic drink**								
Rare/never (329, 32%)	Ref		Ref		Ref		Ref	
1-2 cups/day (683, 67%)	1.02 (1.00, 1.04)	0.10	1.16 (1.11, 1.33)	0.046	0.17 (0.03, 0.31)	0.02	0.15 (0.01, 0.29)	0.04
≥3 cups/day (2, 0.2%)	0.95 (0.93, 0.96)	< 0.001	0.96 (0.94, 0.99)	0.01	0.52 (0.40, 0.64)	< 0.001	0.41 (0.24, 1.42)	< 0.001
*p* for trend		0.15		0.13		0.01		0.03
1 cup/day increment	1.03 (1.00, 1.07)	0.02	1.02 (0.99, 1.05)	0.14	0.18 (0.02, 0.33)	0.02	0.13 (−0.03, 0.30)	0.11

Adjusted models were adjusted by: BMI, energy intake, parental educational level, family income, and household smoking, other drinks (total cups in SSB, fresh juice, fresh milk, flavored milk, soy milk, yogurt, fermented probiotic drink).

### Total and added sugars

**Figure 2:**
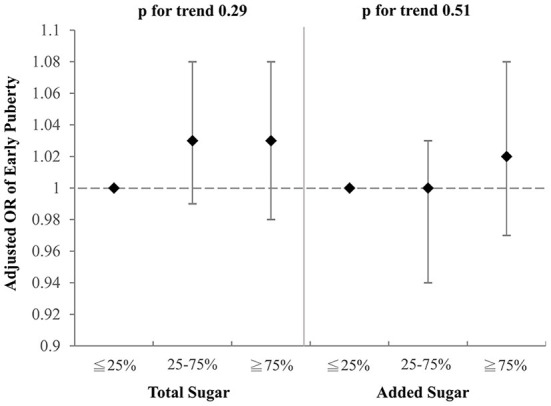
Adjusted OR for early puberty due to the consumption of total sugar and added sugar from selected drinks (boys). Models were adjusted for BMI, parental educational level, family income, and household smoking.

**Figure 3:**
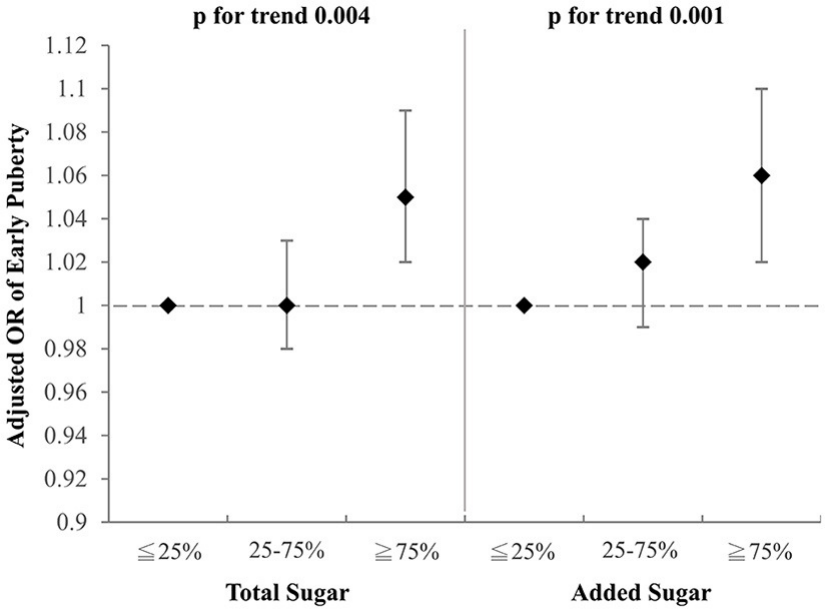
Adjusted OR for early puberty due to the consumption of total sugar and added sugar from selected drinks (girls). Models were adjusted for BMI, parental educational level, family income, and household smoking.

To investigate which component of the selected drinks might contribute to the aforementioned association with early puberty, we examined the effects of total sugar and added sugar from selected drinks on early puberty risk after adjustment for BMI, parental educational level, family income, and household smoking (Figures 2, 3). Notably, among girls, both the intake of total sugar and added sugar predicted an increased likelihood of early puberty in a significant dose–response manner. However, neither total sugar nor added sugar could fully explain the observed associations between more frequent consumption of SSBs and early puberty among boys. Detailed associations between consumption of several sugars and puberty outcomes are listed in Supplementary Tables S1, S2. The intake of total sugar, lactose from natural food, and added sugar each increased the risk of early puberty among girls in a dose–response manner.

## Discussion

### Summary of main findings

In this study, we identified SSB-related risk and protective factors of pubertal timing among Taiwanese adolescents. To our knowledge, this is the first study to reveal that SSB consumption is significantly associated with early puberty in both sexes; probiotic drinks to a higher amount may mitigate this risk. Our findings indicate that health care providers should increase awareness of the effects of SSBs on adolescent health and pubertal development. The novel finding that fermented probiotic drinks or yogurt have a protective effect against early puberty suggests that early puberty might be a gut–brain disease. Further experimental studies are warranted to prove the aforementioned associations.

This is the first study to identify an independent association between SSB consumption and early puberty in boys. In girls, several reports have consistently indicated the association between SSB consumption and early menarche (23, 31, 46). SSB consumption might be associated with metabolic and weight status changes that potentially affect menarcheal timing. The link between obesity and early puberty in girls is well known (38). However, the relationship between obesity and early puberty among boys varies among different ethnicities (35, 47). Several large Chinese studies have reported that obesity also promotes the earlier onset of puberty in boys (47, 48). Furthermore, recent evidence has indicated that SSBs may have a direct effect on sexual maturation in addition to increasing body weight (23). Excessive sugar consumption *per se* is associated with an increase in the production of insulin and IGF-1, which could enhance cell proliferation and growth (49). Evidence also indicates that IGF-1 has a direct effect, through intracellular signal transduction cascades, on the proliferation and differentiation of ovaries as well as testes (50). In summary, excessive SSB consumption may cause fat accumulation and thus increase the risk of obesity, which is a known risk factor for early puberty. Moreover, increased insulin and IGF-1 as a result of excessive sugar intake contribute to dysregulation of the reproductive axis *via* downregulation of SHBG. Further research may be required to identify the key regulator involved in pubertal initiation and how it is linked to sugar metabolism.

We discovered that probiotic drinks appear to be a protective factor against early puberty. This finding is consistent with research (31) that revealed that yogurt delays menarche. We argue that this protection effect may be exerted through modifying dysbiosis in the gut. A paper by Cowan and Richardson (51) indicated that probiotic treatment can restore the normative timing of vaginal opening in stress-induced early-maturing rats. Researchers have also observed that probiotics reverse stress-induced learned fear behavior and stress hormone levels, thus highlighting the restorative effects of probiotics in the context of early-life stress (52). Our findings provide human evidence to further support the crucial role of the gut–brain axis in pubertal development. One prior human study measuring the urine metabolites in patients with central precocious puberty revealed significant alterations in urinary excretion levels of gut microbial-mammalian cometabolites before and after treatment with Triptorelin, suggesting that central precocious puberty may involve an alteration in symbiotic gut microbial composition (53). Given the cumulative evidence of the influence of gut microbiota on the development and functionality of the central nervous system (54), we tentatively conclude that gut microbiota may alter the function of the hypothalamus–pituitary–gonadal axis and thus the activation of puberty. From a practical perspective, changing gut microbiota through the intake of probiotics may attenuate early puberty. On the other hand, although the probiotic content within the yogurt and fermented probiotic drinks might protect children from early puberty, the high sugar contents within these drinks should still be noted. Currently, the World Health Organization (WHO) recommends that children consume foods with low sugar to <50 g/day (55). Hence, if a child consumes more than four cups of yogurt and fermented probiotic drinks, their sugar content within these drinks would exceed the WHO's recommendation. Parents and school policymakers are suggested to provide these probiotic drinks with low or no sugar content for children undergone pubertal growth.

Studies exploring the relationships between milk or diary consumptions and early puberty yielded inconsistent results. Evidence revealed that cow milk was the most commonly consumed animal product by girl, which might influence sexual maturation during adolescence (56, 57). However, a cohort study reported that milk consumption in girls aged ≥9 years failed to predict age at onset of menarche (58). No study has reported the aforementioned association in boys. Although we did not discover significant links between milk consumption and early puberty risk in female participants, consuming flavored milk increased the risk of early puberty in boys. This could be attributed to the extra sugar intake from flavored milk.

The intakes of total and added sugar were associated with an increased risk of early puberty among girls, but not boys. Carwile et al. (23) discovered that added sugar, but not total sugar, was associated with early menarche. Added sugar, such as artificial sweeteners, was characterized to influence metabolic changes and reproductive health by activation of sweet taste receptors (59). Sweet taste receptors locate not only in the mouth cavity but also in ovaries. This was proposed as a possible mechanism why sugar intake may influence the reproductive system. Moreover, routine exposure to added sugars may alter thresholds for sweet taste perception, imparting a stronger preference for sweet food, which may yield adiposity accumulation (60). The detailed mechanism of added sugars on early puberty has not been further explored. The only relevant animal studies reported that long-term *in-utero* exposure of added sugar to 18-day-old mice resulted in a thicker epidermal and dermal layer in the mammary glands of the mice embryos. By the age of 4 weeks after birth, these exposed mice experienced an early production of the mammary gland. This phenomenon indirectly confirmed that long-term exposure to added sugar may prompt early puberty (61).

### Strengths and limitations

The TCHS cohort was nationally representative, systemically sampled, and longitudinally followed; therefore, the derived findings could provide nutritional guidance for our local population. Although same-source bias may be present in a questionnaire-based research design, a detailed FFQ, used to assess food intake, allowed us to calculate nutrient consumption and examine its effect on sexual maturation. However, we did not specify the species and doses of probiotics in our population-based epidemiological study. We were also unable to conclude the direction of the causal relationship between pubertal timing and probiotics use. Given the short time interval in which the exposures and outcomes were assessed (i.e., age 11 years for SSB or probiotic consumption and age 11–12 years for puberty), it is difficult to ascertain whether the exposure truly precedes the pubertal outcome. There could be instances where children in this study had attained puberty before 11 years; this could affect the interpretation of the study findings, which may be biased by reverse causality. Moreover, we tested several regression models in Tables 2, 3 for all exposures and outcomes. We did not specifically adjust for multiple testing, because preferences for food choices were not our primary variables of interest. Despite this, the associations found in the tables remained significant after using stringent corrections for multiple testing. Lastly, the study findings may also be biased by residual confounding from unmeasured covariates, such as water consumption that was not available in the dataset. Further investigation may be required to determine a mechanistic explanation of the association revealed in our study.

## Conclusion

Our study identified an independent association between SSBs and early pubertal timing after adjustments were made for BMI. Probiotic use to a certain amount appeared to mitigate the risk of early puberty. These findings not only highlight the importance of nutritional screening when consulting adolescents going through puberty but also elucidate the gut–brain connection. From the perspective of public health nutrition, a potential way to reduce adverse effects brought by early sexual maturation is to restrict sugar consumption in growing children. More research may be required to identify the specific probiotic species and molecular pathways linking food consumption and pubertal timing.

## Data availability statement

The raw data supporting the conclusions of this article will be made available by the authors, without undue reservation.

## Ethics statement

The studies involving human participants were reviewed and approved by National Taiwan University Ethics Committees. Written informed consent to participate in this study was provided by the participants' legal guardian/next of kin.

## Author contributions

M-CT contributed to data interpretation and writing the manuscript. YCC and YLL contributed to data analysis, cohort data collection, hypothesis generation, data interpretation, and writing the manuscript. All authors approved the final manuscript as submitted and published and agreed to be accountable for all aspects of the work.

## Funding

This study was supported by grants 103-2314-B-002-043-MY3 and 106-2314-B-001-007-MY3 from YLL. The study was also supported by grants 104-2314-B-532-002-MY3, 107-2314-B-038-113-MY3, 109-2314-B-038-057, MOST 110-2314-B-038-156, and MOST 111-2314-B-038-004 (PI. YCC) from the Ministry of Science and Technology of Taiwan.

## Conflict of interest

The authors declare that the research was conducted in the absence of any commercial or financial relationships that could be construed as a potential conflict of interest. The reviewer YCC a shared affiliation with one of the authors' YCC to the handling editor at the time of review.

## Publisher's note

All claims expressed in this article are solely those of the authors and do not necessarily represent those of their affiliated organizations, or those of the publisher, the editors and the reviewers. Any product that may be evaluated in this article, or claim that may be made by its manufacturer, is not guaranteed or endorsed by the publisher.

## Abbreviations

BMI: body mass index
CI: confidence interval
FFQ: food frequency questionnaire
GEE: generalized estimating equation
IGF-1: insulin-like growth factor-1
OR: odds ratio
PDS: Puberty Development Scale
SE: standard error
SSB: sugar-sweetened beverage
TCHS: Taiwan Children's Health Study
TDCS: tanner-derived composite stage.
