# Supplementary material for: Association of the consumption of common drinks with early puberty in both sexes
Source: Front Public Health. 2022 Dec 2;10:854477. doi: 10.3389/fpubh.2022.854477 (PMC9758723; doi:10.3389/fpubh.2022.854477)
Supplement: Supplementary file 1 [file Data_Sheet_1.PDF]

Table S1. OR and regression coefficients for the associations between consumption of sugar and pubertal outcomes (boys)

Category	Early puberty				Age at voice breaking			
	Unadjusted model		Adjusted model		Unadjusted model		Adjusted model	
	OR (95% CI)	P value	OR (95% CI)	P value	β (95% CI)	P value	β (95% CI)	P value
**Total sugar**								
**≦25 (≦41.23 mg)**	ref		ref		ref		ref	
**25%-75% (41.23-67.53 mg)**	1.03 (0.99, 1.08)	0.19	1.03 (0.99, 1.08)	0.20	0.47 (0.08, 0.86)	0.02	0.64 (0.24, 1.03)	0.001
**≧75%(≧67.53 mg)**	1.02 (0.97, 1.07)	0.46	1.03 (0.98, 1.08)	0.30	0.15 (-0.29, 0.59)	0.49	0.35 (-0.10, 0.80)	0.12
**p for trend**		0.45		0.29		0.51		0.13
**1 g/day increment**	1.00 (1.00, 1.00)	0.28	1.00 (1.00, 1.00)	0.19	0.00 (-0.01, 0.00)	0.45	0.00 (-0.01, 0.01)	0.98
**Natural sugar**								
**≦25 (≦8.04 mg)**	ref		ref		ref		ref	
**25%-75% (8.04-23.57 mg)**	1.02 (0.98, 1.07)	0.28	1.02 (0.98, 1.07)	0.39	0.34 (-0.04, 0.72)	0.08	0.50 (0.09, 0.90)	0.02
**≧75% (≧23.57 mg)**	1.00 (0.95, 1.05)	0.98	0.99 (0.95, 1.05)	0.84	0.39 (-0.03, 0.81)	0.07	0.45 (0.01, 0.89)	0.04
**p for trend**		0.94		0.89		0.06		0.04
**1 g/day increment**	1.00 (1.00, 1.00)	0.51	1.00 (1.00, 1.00)	0.53	0.00 (-0.01, 0.01)	0.96	0.00 (-0.01, 0.02)	0.54
**Lactose from natural food**								
**≦25 (≦1.94 mg)**	ref		ref		ref		ref	
**25%-75% (1.94-11.81 mg)**	1.04 (0.99, 1.08)	0.11	1.03 (0.99, 1.08)	0.18	0.50 (0.11, 0.90)	0.01	0.50 (0.09, 0.91)	0.02
**≧75%(≧11.81 mg)**	1.03 (0.98, 1.08)	0.28	1.01 (0.96, 1.06)	0.67	0.37 (-0.08, 0.82)	0.11	0.37 (-0.09, 0.82)	0.11
**p for trend**		0.28		0.66		0.12		0.13
**1 g/day increment**	1.00 (1.00, 1.00)	0.89	1.00 (1.00, 1.00)	0.91	0.00 (-0.02, 0.01)	0.68	0.00 (-0.02, 0.02)	0.90
**Non-lactose from natural food**								
**≦25 (≦1.93 mg)**	ref		ref		ref		ref	
**25%-75% (1.93-11.71 mg)**	1.06 (1.02, 1.11)	0.01	1.05 (1.00, 1.10)	0.03	0.51 (0.12, 0.91)	0.01	0.55 (0.15, 0.95)	0.01
**≧75%(≧11.71 mg)**	1.00 (0.95, 1.05)	1.00	1.01 (0.95, 1.07)	0.74	0.27 (-0.22, 0.77)	0.28	0.44 (-0.06, 0.94)	0.09
**p for trend**		0.85		0.80		0.39		0.12
**1 g/day increment**	1.00 (1.00, 1.00)	0.22	1.00 (1.00, 1.00)	0.48	0.01 (-0.02, 0.03)	0.58	0.02 (-0.01, 0.05)	0.18
**Added sugar**								
**≦25 (≦28.55 mg)**	ref		ref		ref		ref	
**25%-75% (28.55-44.83 mg)**	0.99 (0.95, 1.04)	0.79	1.00 (0.94, 1.03)	0.60	0.24 (-0.17, 0.65)	0.26	0.15 (-0.27, 0.57)	0.49

**≧75% (≧44.83 mg)**	1.02 (0.97, 1.07)	0.51	1.02 (0.97, 1.08)	0.41	0.11 (-0.35, 0.57)	0.64	0.16 (-0.30, 0.62)	0.49
**p for trend**		0.51		0.51		0.66		0.49
**1 g/day increment**	1.00 (1.00, 1.00)	0.07	1.00 (1.00, 1.00)	0.04	0.00 (-0.01, 0.00)	0.26	0.00 (-0.01, 0.01)	0.67

Adjusted models were adjusted by: BMI, parental educational level, family income, and household smoking

Table S2. OR and regression coefficients for the associations between consumption of sugar and pubertal outcomes (girls)

Category	Early puberty				Age at menarche			
	Unadjusted model		Adjusted model		Unadjusted model		Adjusted model	
	OR (95% CI)	P value	OR (95% CI)	P value	β (95% CI)	P value	β (95% CI)	P value
**Total sugar**								
**≦25 (≦41.23 mg)**	ref		ref		ref		ref	
**25%-75% (41.23-67.53 mg)**	1.00 (0.97, 1.03)	0.96	1.00 (0.98, 1.03)	0.71	-0.004 (-0.16, 0.15)	0.96	0.08 (-0.17, 0.16)	0.97
**≧75%(≧67.53 mg)**	1.05 (1.02, 1.09)	0.005	1.05 (1.02, 1.09)	0.004	0.17 (-0.01, 0.35)	0.06	0.09 (0.00, 0.37)	0.047
**p for trend**		0.005		0.004		0.07		0.048
**1 g/day increment**	1.00 (1.00, 1.00)	0.02	1.00 (1.00, 1.00)	0.030	0.003 (0.001, 0.01)	0.01	0.001 (0.001, 0.01)	0.01
**Natural sugar**								
**≦25 (≦8.04 mg)**	ref		ref		ref		ref	
**25%-75% (8.04-23.57 mg)**	1.01 (0.98, 1.04)	0.56	1.01 (0.98, 1.04)	0.68	0.002 (-0.15, 0.16)	0.98	0.08 (-0.17, 0.16)	0.92
**≧75% (≧23.57 mg)**	1.01 (0.97, 1.04)	0.70	1.01 (0.97, 1.04)	0.64	0.22 (0.05, 0.39)	0.01	0.09 (0.06, 0.41)	0.01
**p for trend**		0.71		0.64		0.01		0.01
**1 g/day increment**	1.00 (1.00, 1.00)	0.27	1.00 (1.00, 1.00)	0.25	0.004 (-0.001, 0.01)	0.08	0.003 (0.00, 0.01)	0.07
**Lactose from natural food**								
**≦25 (≦1.94 mg)**	ref		ref		ref		ref	
**25%-75% (1.94-11.81 mg)**	1.02 (1.00, 1.05)	0.06	1.02 (1.00, 1.05)	0.08	-0.02 (-0.17, 0.13)	0.76	0.08 (-0.19, 0.12)	0.65
**≧75%(≧11.81 mg)**	1.04 (1.01, 1.08)	0.014	1.04 (1.00, 1.07)	0.04	0.09 (-0.09, 0.27)	0.31	0.09 (-0.11, 0.26)	0.42
**p for trend**		0.01		0.03		0.35		0.47
**1 g/day increment**	1.00 (1.00, 1.00)	0.09	1.00 (1.00, 1.00)	0.12	0.01 (-0.001, 0.01)	0.08	0.003 (-0.001, 0.01)	0.11
**Non-lactose from natural food**								
**≦25 (≦1.93 mg)**	ref		ref		ref		ref	
**25%-75% (1.93-11.71 mg)**	1.00 (0.96, 1.03)	0.89	1.00 (0.97, 1.04)	0.82	0.11 (-0.08, 0.31)	0.24	0.10 (-0.09, 0.31)	0.29
**≧75%(≧11.71 mg)**	0.99 (0.95, 1.03)	0.66	1.00 (0.96, 1.04)	0.93	0.16 (-0.06, 0.37)	0.15	0.12 (-0.07, 0.39)	0.17
**p for trend**		0.62		0.85		0.18		0.19
**1 g/day increment**	1.00 (1.00, 1.00)	0.93	1.00 (1.00, 1.00)	0.80	0.00 (-0.01, 0.02)	0.37	0.01 (-0.01, 0.02)	0.34
**Added sugar**								
**≦25 (≦28.55 mg)**	ref		ref		ref		ref	
**25%-75% (28.55-44.83 mg)**	1.01 (0.99, 1.04)	0.27	1.02 (0.99, 1.04)	0.24	0.19 (0.04, 0.35)	0.02	0.08 (0.05, 0.37)	0.01
**≧75% (≧44.83 mg)**	1.06 (1.02, 1.09)	0.001	1.06 (1.02, 1.10)	0.001	0.28 (0.10, 0.46)	0.003	0.09 (0.11, 0.48)	0.002

**p for trend**		0.001		0.001		0.002		0.002
**1 g/day increment**	1.00 (1.00, 1.00)	0.02	1.00 (1.00, 1.00)	0.02	0.005 (0.001, 0.01)	0.02	0.00 (-0.01, 0.01)	0.02

Adjusted models were adjusted by: BMI, parental educational level, family income, and household smoking

Table S3. Contents of Yoghurt and Fermented Probiotic Drinks assessed in Taiwan Children Health Study

**Drinks**	**Yogurt**	**Fermented Probiotic Drinks**		
		**Yakult**	**Fermented milk**	**Probiotic drinks**
**Probiotics content**	*Streptococcus thermophiles*<br>*Lactobacillus acidophilus* La-5<br>*Bifidobacterium lactis* Bb-12<br>*Lactobacillus bulgaricus*	*L. casei Shirota*	Lactobacillus	Lactobacillus paracasei<br>Lactobacillus helveticus
**Sugar**	11.5g/100ml	13.6g/100ml	12.9g/100ml	13.1g/100ml
**Protein**	3g/100ml	1.2g/100ml	1g/100ml	1.1g/100ml
